# Use of Mealworm (*Tenebrio molitor*) Flour as Meat Replacer in Dry Fermented Sausages

**DOI:** 10.3390/foods14061019

**Published:** 2025-03-17

**Authors:** Xavier F. Hospital, Eva Hierro, Manuela Fernández, Diana Martin, Rosa Escudero, Joaquín Navarro del Hierro

**Affiliations:** 1 Sección Departamental de Tecnología Alimentaria, Facultad de Veterinaria, Universidad Complutense de Madrid, 28040 Madrid, Spain; hierro@ucm.es (E.H.); manuela@ucm.es (M.F.); 2 Sección Departamental de Ciencias de la Alimentación, Facultad de Ciencias, Universidad Autónoma de Madrid, 28049 Madrid, Spain; diana.martin@uam.es; 3 Departamento de Producción y Caracterización de Nuevos Alimentos, Instituto de Investigación en Ciencias de la Alimentación (CIAL) (CSIC–UAM), 28049 Madrid, Spain; 4 Departamento de Producción Animal, Facultad de Veterinaria, Universidad Complutense de Madrid, 28040 Madrid, Spain; rmescude@ucm.es

**Keywords:** mealworm flour, dry fermented sausages, edible insects, sustainable protein, novel food, lipid oxidation

## Abstract

The increasing demand for sustainable and nutritionally rich protein sources has led to a growing interest in edible insects as a viable alternative to traditional meat. This study evaluates the potential of mealworm (*Tenebrio molitor*) flour as a partial meat replacer in the formulation of dry fermented sausages (salchichón). Four formulations were prepared, replacing 0%, 5%, 10%, and 15% of pork meat with mealworm flour, and their microbiological, physicochemical, rheological, technological, and sensory properties were analyzed. Results showed that the incorporation of mealworm flour did not compromise the growth of lactic acid bacteria or Gram-positive catalase-positive cocci, both essential for fermentation and curing. The inclusion of mealworm flour significantly increased the protein, fiber, and polyunsaturated fatty acid (PUFA) content of the sausages, improving their nutritional profile. Notably, despite the higher PUFA content, lipid oxidation was reduced, as evidenced by lower concentrations of oxidation-derived volatile compounds. Significant changes were also observed in color, particularly at higher replacement levels, which resulted in a noticeable darkening of the sausages. Sensory evaluation indicated that replacing up to 5% of pork meat maintained product acceptability, whereas higher levels caused significant changes. The partial replacement of pork by mealworm flour shows interesting possibilities to produce more sustainable and functional dry fermented meats.

## 1. Introduction

The increasing global demand for meat and meat products has led to significant environmental and ethical concerns. The intensive livestock farming required to meet this demand contributes to greenhouse gas emissions, deforestation, and excessive water usage [1]. Additionally, the growing awareness of animal welfare issues has prompted consumers and researchers to seek sustainable and ethical alternatives to conventional meat.

To address these challenges, various alternatives have been proposed, including plant-based and microbial proteins, cultured meat, and edible insects. The latter have emerged as a particularly promising option since edible insects are not only rich in high-value proteins but also contain essential vitamins, minerals, and fatty acids [2]. In terms of environmental sustainability, insects offer significant advantages over traditional livestock. They require substantially less land and water for production, have a high feed conversion efficiency, and their farming results in lower greenhouse gas emissions [3]. Efficiency, combined with their reduced environmental footprint, makes edible insects a viable and sustainable alternative protein source.

In the European Union (EU), the regulatory framework for the use of insects in food has evolved. The European Food Safety Authority (EFSA) has conducted risk assessments for several insect species, including mealworm (*Tenebrio molitor*), and has deemed them safe for human consumption under certain conditions [4]. Mealworm is characterized by its ease of breeding and high nutritional value, with a protein content comparable to that of traditional animal sources [5], making it a potential ingredient for fortifying foods and improving their nutritional profile. Additionally, its lipid fraction is characterized by a favorable ratio of unsaturated to saturated fatty acids [6], which may have beneficial health implications. Thus, its approval as a novel food in the EU represents a significant step towards the mainstream acceptance of insects as a viable food source [7]. Consequently, there has been growing interest in incorporating mealworm flour into various food products, including baked goods, snacks, meat analogs, and meat products [8,9,10,11,12].

Previous studies have primarily explored the incorporation of mealworm and other insect flours into raw and cooked meat products, highlighting their potential to enhance their nutritional profile, although they also influence their physicochemical, rheological, and sensory properties at certain concentrations [11,12,13,14,15,16,17,18]. However, research on the incorporation of insect flour into dry fermented meat products remains limited. Salchichón, a traditional Spanish sausage typically made with pork meat and fat, is seasoned with black pepper as a characteristic spice and undergoes fermentation and drying processes that shape its distinct sensory profile.

This study aims to evaluate the effects of partially replacing pork meat with mealworm flour on the microbiological, physicochemical, rheological, technological, and sensory properties of salchichón.

## 2. Materials and Methods

### 2.1. Mealworm Flour Preparation

Dried larvae of mealworm (*T. molitor*) were obtained from Nimavert (Meise, Belgium) and stored at 4 °C until their use. The mealworms were ground in a blender for 2 min to achieve a particle size < 0.5 mm. The nutritional composition of the larvae (g/100 g), as provided by the manufacturer, was protein (50), fat (31), saturated fat (7.6), carbohydrates (6.7), sugars (<0.2), dietary fiber (3.3), and NaCl (0.3).

### 2.2. Sausage Manufacture

Semi-frozen pig leg meat (lean) and pork back fat (QuiroCarne S.L., Leganés, Spain) were minced separately to a 6 mm particle size. Four different experimental formulations were prepared with 56–70% lean by replacing 0 (control), 5, 10, or 15% of lean meat with mealworm flour. Subsequently, the rest of the ingredients (Anvisa, Madrid, Spain) were added (pork back fat, lactose, NaCl, ice water, ground black pepper, NaNO_2_, NaNO_3_, and sodium ascorbate), as shown in Table S1. To compensate for the moisture of the replaced lean, formulations with insect flour were supplemented with the corresponding amount of water. A cocktail of *Staphylococcus xylosus* S-SX (Christian Hansen, Hoersholm, Denmark), *Staphylococcus carnosus* CECT 4491 (Colección Española de Cultivos Tipo, CECT, Valencia, Spain), and *Latilactobacillus sakei* isolated from an artisan sausage was added to achieve approximately 6 log cfu/g for staphylococci and 7 log cfu/g for lactobacilli.

After a mixing step, the different mixtures were stuffed into 45 mm collagen casings to obtain sausages of approximately 200 g. The fresh sausages were superficially sprayed with a 20% potassium sorbate solution to prevent mold growth. Afterwards, sausages were ripened for 30 days in a Binder KMF 115 chamber (Tuttlingen, Germany) under the following conditions: 48 h at 22 °C and 90% relative humidity (RH), 24 h at 19 °C and 88% RH, 24 h at 15 °C and 86% RH, and 26 days at 12 °C and 85% RH. At the end of ripening, two sausages per batch were sampled for duplicate analysis. Two independent productions of dry fermented sausages were carried out on different days.

### 2.3. Analysis of the Typical Microbiota of Sausages

Ten-gram samples were aseptically transferred to a Stomacher bag and homogenized with 50 mL of sterile saline solution (0.85% NaCl) using a Tekmar Stomacher Lab Blender STO-80 (Bedfordshire, UK) for 2 min. Then, the homogenate underwent serial 10-fold dilutions, and 0.1 mL of each appropriate dilution was plated onto agar plates.

Lactic acid bacteria (LAB) were enumerated on double-layer pH 5.5 MRS agar (Pronadisa, Madrid, Spain) after incubation at 32 °C for 48 h. Gram-positive catalase-positive cocci (GCC+) were cultured on mannitol salt agar (MSA, Pronadisa, Madrid, Spain) at 32 °C for 48 h. *Enterobacteriaceae* were enumerated on double-layer Violet Red Bile Glucose (VRBG, Pronadisa, Madrid, Spain) agar with incubation at 37 °C for 24 h. The results were expressed as log cfu/g of sample.

### 2.4. pH and Water Activity

The pH measurement was conducted using a Hanna HI98161 pH meter equipped with a penetration electrode FC2023 (Hanna Instruments, Woonsocket, RI, USA). Water activity (a_w_) was assessed in 3 mm thick sausage slices using a dew point hygrometer AquaLab 4TE (Meter Group, Pullman, WA, USA) at 25 °C.

### 2.5. Proximate Composition

Moisture, crude protein (N *×* 6.25), crude fiber, and ash and fat content (hexane extract) of the sausages were determined using the standard methods described by the Association of Official Analytical Chemists [19]. Carbohydrates were calculated from the difference according to Equation (1). The results were expressed as g/100 g of dry matter.

(1) Carbohydrates (g/100 g)=100−(% Protein+% Fat+% Crude fiber+% Ash)

### 2.6. Chemical Determinations

#### 2.6.1. Fatty Acid Profile

Fat was obtained by a two-step extraction with hexane as the non-polar solvent according to Bußler et al. [20]. Briefly, a solid-to-solvent ratio of 1:5 with hexane was blended in an Ultra-Turrax T18 homogenizer (Ika, Staufen, Germany). Subsequently, the homogenate was centrifuged at 1900× *g* for 10 min and the precipitate was subjected to a second extraction cycle. Both supernatants were combined and evaporated using a rotary evaporator (Büchi Labortechnik R-200, Flawil, Switzerland).

FA analysis was conducted according to the protocol by Otero et al. [21]. Samples of 5 mg were saponified with 250 µL of a solution prepared with 45 g NaOH in 150 mL of distilled water and 150 mL of methanol. After 30 min of incubation at 100 °C, the FAs were methylated with 500 µL of 6 N HCl in methanol, followed by heating in a water bath at 80 °C for 20 min. Upon cooling, FA methyl esters (FAMEs) were extracted using a mixture of hexane and anhydrous diethyl ether (1:1, *v*/*v*), followed by centrifugation at 13,200× *g* for 5 min. The upper phase was recovered and dried under nitrogen flow. Then, the extract was dissolved in 150 µL hexane and injected (1 μL in splitless mode) into a gas chromatograph Agilent 7890A coupled with an FID detector (Agilent Technologies, Loveland, CO, USA). Separation was achieved with an HP-5MS UI capillary column (30 m × 0.250 mm × 0.25 μm, Agilent). The GC oven temperature started at 50 °C, then increased to 210 °C at a 20 °C/min rate and held for 18 min. Subsequently, the temperature was raised to 230 °C at 20 °C/min and held for 13 min.

The identification and quantification of FAs were carried out using individual calibration curves constructed with commercial standards. Results were expressed as the percentage of total FAs. The hypocholesterolemic/hypercholesterolemic (h/H) ratio was calculated according to Equation (2) [22]:

(2) $$h/H=\frac{\left(C18:1n9c+C18:2n6c+C20:2n6+C20:3n6++C20:4n6+C22:4n6+C22:5n3\right)}{\left(C12:0++C14:0+C16:0\right)}$$

#### 2.6.2. Determination of Lipid Hydroperoxides and Thiobarbituric Acid-Reactive Substances (TBARs)

Primary and secondary lipid oxidation products were determined according to Hospital et al. [23]. Lipid hydroperoxides, as an indicator of primary oxidation, were measured by mixing 2 g of sausage with 25 mL of chloroform/methanol (1:1, *v*/*v*) and 0.5 mL of a 0.19 M BHT ethanolic solution. The mixture was homogenized under cold conditions at 7000 rpm for 2 min using an Ultra-Turrax T18 homogenizer (Ika, Staufen, Germany). Following the addition of 6 mL of distilled water, samples were centrifuged at 3000× *g* for 30 min at 5 °C, and the chloroform/methanol layer was removed and vigorously mixed with 2.8 mL of methanol/1-butanol (2:1, *v*/*v*). Then, 15 μL of a ferrous iron solution and 15 μL of 3.94 M ammonium thiocyanate were added. The mixture was left in darkness for 20 min at room temperature, and the absorbance was measured at 510 nm using a spectrophotometer U-2000 (Hitachi, Tokyo, Japan). The concentration of hydroperoxides was determined using a standard curve of cumene hydroperoxide (0–20 μM), and the results were expressed as mmol hydroperoxide/kg of the sample.

Secondary oxidation products were assessed by measuring the thiobarbituric acid-reactive substances (TBARs). Two grams of the sample was blended for 2 min in an Ultra-Turrax T18 homogenizer (Ika, Staufen, Germany) with 8 mL of 5% trichloroacetic acid (TCA) and 0.5 mL of a 0.19 M BHT ethanolic solution. The resulting homogenate was filtered, adjusted to a volume of 10 mL with 5% TCA, and 700 µL of the filtrate was mixed with 0.7 mL of 20 mM thiobarbituric acid. The reaction was conducted in a water bath at 100 °C for 30 min, and the absorbance was measured at 532 nm. Malondialdehyde (MDA) concentration was determined using a standard curve of 1,1,3,3-tetramethoxypropane (0–10 μM). The results were expressed as mg of MDA/kg of the sample.

#### 2.6.3. Volatile Compound Analysis

Solid-phase microextraction (SPME) followed by GC-MS analysis was used to identify and quantify the headspace volatiles from the sausages. For this purpose, a portion of 4 g of sausage previously ground was transferred to a 15 mL headspace glass vial sealed with a PTFE/silicone septum (Supelco, Bellefonte, PA, USA), which was then placed on a CombiPAL autosampler (Agilent Technologies, Santa Clara, CA, USA). Volatile compounds were adsorbed onto a carboxen/polydimethylsiloxane (CAR/PDMS) fiber (1 cm, 85 μm; Supelco Inc., Bellefonte, PA, USA) for a 30 min period, while the sample was maintained at 40 °C following equilibration at the same temperature for 30 min. Subsequent to adsorption, the compounds were desorbed in a gas chromatograph Agilent 8890 coupled with an Agilent 7000D triple quadrupole mass spectrometer (Agilent Technologies, Santa Clara, CA, USA). Separation was achieved using a 5MS/silphenylene polysiloxane fused silica capillary column (60 m × 0.25 mm i.d., 1 μm film thickness, Quadrex Corporation, Woodbridge, CT, USA). The GC oven program was initiated at 40 °C for 3 min, increased to 280 °C at 4 °C/min, and held for 5 min. To determine the linear retention index (LRI) values for each component, a C8-C20 n-alkane standard solution (Sigma, St. Louis, MO, USA) was analyzed under identical conditions. Compound identification involved comparing mass spectra with those of authentic standards and/or those in the NIST20 (National Institute of Standards and Technology, MD, USA) Mass Spectral Library. When possible, identities were confirmed by comparing LRI values with either commercial standards or published values [24,25]. Peak areas were integrated using Mass Hunter workstation software 10.0 (Agilent). Results were expressed in area units ×10^−5^.

### 2.7. Color Analysis

The color parameters L* (lightness), a* (redness), and b* (yellowness) were measured according to the CIELAB color space. Sausages were cut into 10 mm thick slices and color was immediately measured at four randomly chosen points using a tristimulus colorimeter (ChromaMeter CR-400, Konica Minolta Sensing, Osaka, Japan) with an 8 mm measuring diameter. Given the heterogeneity of the samples, measurements were taken exclusively on the lean portion of the slices to ensure consistency and minimize variability. All measurements were performed against a white background to standardize potential background interference across all formulations. The total color difference (ΔE*) for each sample relative to the control sausage was calculated using Equation (3):

(3) $$\Delta E^{*}=\sqrt{(\Delta L^{*}{)}^{2}+(\Delta a^{*}{)}^{2}+(\Delta b^{*}{)}^{2}}$$

### 2.8. Texture Analysis

A texture profile analysis (TPA) was conducted on sausage cylinders, each measuring 1 cm in height and 3 cm in diameter after removing the casings. A double compression cycle test was performed using a Texture Analyser TA.XT2i (Stable Microsystems, Surrey, UK) equipped with Texture Expert software 6.2.6. The test involved compressing the original portion by up to 50% height with a 5 s pause between the end of the first cycle and the start of the second one. The compression was applied using an aluminum cylinder probe P/25 (25 mm diameter) at room temperature. Force–time deformation curves were recorded with a 25 kg load cell applied at a crosshead speed of 2 mm/s. Hardness (N), springiness, adhesiveness (N × s), cohesiveness, and chewiness (N) were determined according to Bourne [26].

### 2.9. Sensory Analysis

A sensory evaluation was conducted to compare the different formulations of salchichón using a rank-order test, a method that assesses perceptible differences among product samples. The evaluation was performed by 18 panelists familiar with the sensory characteristics of dry fermented meat products. Panelists were selected based on their prior experience with fermented meats but did not undergo specific sensory training for this study. To prevent bias in the sensory evaluation, panelists were not informed about the specific composition of the samples.

The sensory analysis was conducted in an ISO-standardized tasting room [27]. Panelists were not informed that some sausages contained mealworm flour, nor were they aware of the control batch identity. However, due to potential allergenic risks, participants were advised to refrain from the test if they had known allergies to crustaceans, mites, or mollusks.

Each panelist evaluated the four different samples based on odor, color, texture, and taste, using a rank-order scale, where 1 represented the least preferred sample and 4 the most preferred (no two samples could receive the same score). The sum of ranks (SR) for each attribute was calculated according to the methodology described by Fernández et al. [28].

### 2.10. Statistical Analysis

Statgraphics Centurion 19 (Statpoint Technologies, Warrenton, VA, USA) was used for the statistical analysis. A one-way ANOVA test was conducted to compare the results obtained from the different formulations. Statistical significance was identified at a 95% confidence level. Tukey’s multiple range tests were used to assess differences between pairs of means.

For sensory analysis, the significance level of data obtained in the rank order test was determined by Friedman’s rank addition according to the tables of “all treatments”, a multiple comparison for the analysis of ranked data [29].

## 3. Results and Discussion

### 3.1. Microbiological and Physicochemical Characterization

The initial numbers of LAB and GCC+ in the sausages were 7.0 and 5.6 log cfu/g, respectively. The addition of a starter culture at high concentrations is crucial to ensure the rapid establishment of the desired microbial community, which plays an essential role in the fermentation process by promoting color and flavor development and contributing to product safety through the inhibition of other microorganisms. By the end of the ripening process, LAB reached levels of 8.6–9.0 log cfu/g, with no significant differences observed between formulations. Regarding GCC+, these microorganisms exhibited growth to final levels of 7.0–8.0 log cfu/g, with significant differences up to 1 log observed between the formulations with 0–5% and 10–15% of mealworm flour (Table 1). These results indicate that mealworm flour does not negatively impact the growth of the technological microbiota. The presence of GCC+ is particularly important due to their technological roles, including color development, proteolysis, and lipolysis, all of which contribute to the sensory properties of fermented sausages. Similar final numbers of *Micrococcaceae* were described by other authors in conventional fermented sausages [30,31]. Additionally, De Smet et al. [32] demonstrated that mealworm paste served as an excellent substrate for fermentation when inoculated with a commercial meat starter culture comprising *Pediococcus acidilactici*, *Lactobacillus curvatus*, and *S. xylosus*. Similarly, Borremans et al. [33] reported that *L. sakei* and other LAB exhibited effective fermentation activity when also applied to mealworm paste.

The inclusion of the insect flour did not lead to significant differences in pH and a_w_, with similar values recorded in all formulations (Table 1). In this respect, the pH values of the sausages were 5.3–5.4, while a_w_ values varied between 0.84 and 0.86. These parameters are crucial for the stability and safety of dry-cured sausages, as low pH and a_w_ inhibit the growth of competitive microorganisms. In our case, despite the successful development of LAB, the decrease in pH was less pronounced, since typical values reported in Mediterranean dry fermented sausages generally fall below pH 5.0 [34]. The higher pH found in our study could be attributed to a slightly more intense proteolysis that various microorganisms may cause, including GCC+ [30]. Nevertheless, the pH values observed were within the range reported for other traditional fermented sausages [30,35].

Lastly, *Enterobacteriaceae* levels were below the limit of detection (1.5 log cfu/g), with no significant differences observed between treatments (Table 1). These values indicate a satisfactory microbiological quality for this kind of product [36].

### 3.2. Proximate Composition

Moisture content increased significantly with increasing concentrations of mealworm flour (*p* < 0.05), ranging from 24.3 to 28.0 g/100 g for the 0% and 15% formulations, respectively (Table 1). It has been reported that porcine myofibrillar proteins form more stable structures capable of trapping water molecules more efficiently than insect proteins [37]. Despite this, in our study, insect flour might have contributed to moisture retention. The effect of edible insects on water-holding capacity and moisture loss reported in the literature are variable. Cavalheiro et al. [18] observed that the use of cricket flour to replace meat protein decreased moisture content in cooked sausages. Kim et al. [37] reported that replacing porcine myofibrillar protein with mealworm protein reduced the water-holding capacity of emulsion systems, which was attributed to protein denaturation during the drying process of the insects. However, other authors have reported an increasing effect of non-meat proteins on water-holding capacity [38].

Similarly, the protein, fat, and total fiber content, expressed on a dry matter basis, also showed significant variations between formulations. The increase in protein reflects the higher levels provided by the mealworm flour, consistent with the findings of Choi et al. [39] and Cavalheiro et al. [18], who reported a gradual rise in this component with the addition of mealworm or cricket flour in frankfurters. The significant rise in total fiber content can also be attributed to the mealworm flour, which contained 3.3 g/100 g of fiber, and the absence of fiber in the meat. Thus, the fiber content was more prominent as the proportion of pork was reduced. These results align with the expectation that insect flours, being rich in proteins and dietary fiber, could contribute to the overall protein and fiber content of the final product, especially at higher inclusion levels.

Conversely, despite the higher fat content of mealworm flour compared to pork lean meat, a significant decrease in fat levels was observed with increasing concentrations of mealworm flour. Similar findings have been reported in emulsion sausages formulated with this ingredient [39]. In contrast, the inclusion of mealworm flour had no significant effect on the ash content and carbohydrates, which remained comparable to that of the control formulation.

### 3.3. Fatty Acid Profile

The incorporation of mealworm flour into sausages resulted in a limited effect on the FAME profile, with some significant changes in the content of certain individual fatty acids, as detailed in Table 2. Specifically, myristic acid (C14:0) and linoleic acid (C18:2n-6c) increased significantly with meat replacement, while heptadecenoic acid (C17:1n-7), heptadecanoic acid (C17:0), nonadecanoic acid (C19:1n-9), and eicosadienoic acid (C20:2n-6) exhibited an opposite behavior.

The observed increase in C18:2n-6c is consistent with previous studies that have identified this fatty acid, along with palmitic (C16:0) and oleic acids (C18:1n-9c), as one of the most abundant fatty acids in whole mealworms [31,40,41]. The content of C18:2n-6c in mealworms is notably higher than that typically found in conventional animal-based products. This is particularly beneficial from a nutritional perspective, as C18:2n-6c is an essential fatty acid required in mammalian diets and must be obtained through food consumption [32].

The overall changes in individual fatty acids primarily affected the total PUFA content, which was the lowest in the formulation without mealworm flour. This was reflected in a higher PUFA/SFA ratio in mealworm formulations (*p* < 0.05). This is nutritionally significant, as PUFAs are known for their health benefits, including improved cardiovascular health. However, no significant differences were observed for the h/H ratio. It is important to note that the relative proportions of fatty acids within each insect species can widely vary due to environmental factors, such as the type of substrate on which they are fed [42]. Therefore, standardizing rearing conditions is essential to achieve a more consistent overall lipid composition in insects and their products.

### 3.4. Oxidative Stability

Peroxide values and the TBAR index were measured to assess the oxidative stability of the sausages (Table 1). Peroxides ranged from 38.9 to 51.2 mmol hydroperoxides/kg. Although these compounds do not directly contribute to aroma, they act as precursors for the formation of odor active compounds in secondary reactions. Regarding TBARs, the values were 3.3–3.9 mg MDA/kg. No significant differences were observed in both parameters among the formulations, indicating that mealworm flour did not adversely affect lipid oxidation within the tested concentrations. This is noteworthy, as it has been previously mentioned that the fatty acid profile of mealworm formulations was richer in PUFAs (Table 2). This oxidative stability could be attributed to the presence of carotenoids, flavonoids, and other phenolic compounds in insects, which are known for their antioxidant properties [43].

These results are in agreement with those reported by Kim et al. [13], who evaluated the effect of whole mealworm flour on the lipid oxidation of cooked emulsion sausages after 7 days of exposure to continuous fluorescent natural white light at 2 °C. These authors found that the inclusion of 10% of this flour resulted in TBAR values similar to a conventional sausage, suggesting that mealworm flour would not accelerate lipid oxidation in the product.

### 3.5. Volatile Profile

The headspace volatile profile of dry fermented sausages containing different concentrations of mealworm flour is shown in Table 3. A total of 59 volatile compounds were identified, semi-quantified as area units, and classified according to their most likely origin. Significant differences (*p* < 0.05) were observed in the total volatile content between the control batch and the formulations with 10 and 15% mealworm flour, which exhibited a higher content of volatile substances. Furthermore, notable variations were found in the concentration of several individual volatiles, which is particularly relevant, as the abundance of these compounds play a key role in defining the product’s aroma [44].

Volatiles derived from carbohydrate fermentation were the most abundant group in sausages formulated with mealworm flour. The levels of these volatiles rose as the concentration of flour increased, accounting for 54, 64, and 71% of the total volatiles for 5, 10, and 15% formulations, respectively, while this fraction only represented 26% in the control sausages. Significant differences (*p* < 0.05) were found among all batches (Table 3).

Acetic acid, a potent odorant in fermented sausages, was the dominant compound in this group in all formulations, and its concentration significantly increased with the addition of mealworm flour. Acetic acid is also the main volatile compound found in many insects. In the specific case of mealworm flour, Perez-Santaescolastica et al. [45] reported that acetic acid accounted for half of the total volatile compounds, although other authors have reported lower levels [46]. Therefore, part of the acetic acid quantified in the reformulated sausages could originate from the insect flour itself, although this alone would not fully explain the total amount of this compound present in the experimental sausages. Other compounds, such as 3-hydroxy-2-butanone (acetoin) and 2,3-butanedione, both contributing to a buttery aroma, followed a similar behavior to acetic acid. All of these volatile compounds mainly originate from the metabolic activity of microorganisms on carbohydrates, which in fermented sausages is primarily attributed to LAB and GCC+ [47]. Accordingly, the lower levels of carbohydrate-derived volatiles observed in the control batch could be explained by the 1 log cfu/g lower counts of GCC+. The influence of the typical microbiota on the volatile profile of fermented sausages has been previously reported [48,49,50].

Volatiles resulting from lipid oxidation accounted for 3–32% of the total volatile compounds and were significantly affected (*p* < 0.05) by the addition of mealworm flour. This was the major fraction in the control sausages, which exhibited a significantly higher (*p* < 0.05) concentration (7–8-fold) compared to the reformulated sausages. No significant differences were found among mealworm formulations. These results suggest that the insect flour reduced lipid oxidation, which as previously mentioned could be attributed to the presence of pigments with antioxidant activity. Aliphatic aldehydes are considered one of the most important chemical groups in fermented sausages due to their significant contribution to aroma, specifically to green and rancid aroma notes [44]. Notably, hexanal and pentanal, which are commonly detected at high concentrations in fermented sausages [51], were markedly reduced even with the replacement of just 5% of pork meat with mealworm flour. The differences observed in lipid oxidation volatiles were not reflected in the results of the oxidation parameters, and particularly in TBARs, which accounts for secondary oxidation, though higher values (although not significant) were found in the control batch (Table 1).

Conversely, two specific compounds within the lipid oxidation group, 2,2,4,6,6-pentamethylheptane and 2-hydroxy-3-pentanone, showed an increase in concentration with higher levels of mealworm flour. These compounds were absent in the control group, suggesting that they likely originated from the insect flour. 2,2,4,6,6-pentamethylheptane is a hydrocarbon that has been previously detected in insects, including mealworm [45].

The degradation of amino acids represented the origin of 2–5% of the total extracted volatiles. Significant differences (*p* < 0.05) were observed in this group of compounds among the sausages, with the control batch exhibiting higher levels compared to those made with insect flour. In fermented products, these compounds are produced by the activity of GCC+ and LAB starter cultures, which catabolize the branched-chain amino acids leucine, valine, and isoleucine to branched-chain aldehydes (2-methylbutanal and 3-methylbutanal) and their corresponding alcohols (2-methyl-1-butanol and 3-methyl-1-butanol) and acids (3-methylbutanoic acid), all of which contribute to the characteristic matured aroma of cured meat products [52]. Although the control sausages showed lower levels of GCC+, the higher concentrations of these volatiles could be partly explained by a higher presence of meat proteases in this batch, since proteolytic phenomena in dry sausages are mainly due to endogenous enzymes [53]. The contribution of insect proteolytic activity in sausages containing mealworm flour may have been limited, as their endogenous enzymes could have been inactivated during the processing of the flour. Treatments such as drying and grinding, which are commonly applied to prepare insect flour, often involve high temperatures or mechanical stress that can denature proteins and inactivate enzymatic systems. Furthermore, in the sausages prepared with mealworm flour, the main compound of this group, 3-methylbutanal, might have been transformed into other derivatives such as 3-methylbutanoic acid, whose content was significantly higher in these sausages.

Regarding the esters and terpenes identified, which originate from microbial esterification and spices, respectively, no clear correlation was observed between their levels and the concentration of mealworm flour incorporated. Ethyl acetate was the predominant ester, contributing to the fruity and caramel notes characteristic of fermented sausages [51]. Terpenes, on the other hand, were associated with vegetal aroma notes, including resin, pine, lemon, and menthol [44].

### 3.6. Color Analysis

**Figure 1:**
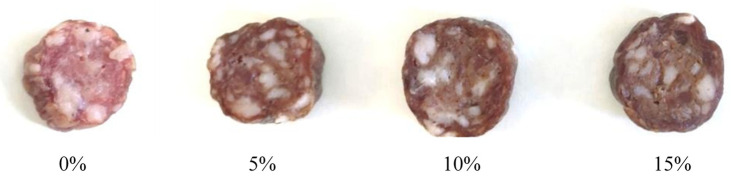
Visual appearance of slices of fermented sausages made with increasing concentrations of mealworm flour in substitution of pork meat.

The impact of varying concentrations of mealworm flour on the CIELAB color parameters was evaluated, as changes in color can significantly influence the primary consumer perception. While the a* parameter was not significantly affected by the formulation, a significant reduction in L* was observed with increasing concentrations of mealworm flour (Table 4), indicating a noticeable darkening of the sausages with higher levels of meat replacement. In contrast, b* increased significantly with higher flour concentrations, with values ranging from 5.5 (0%, control) to 9.6 (15% flour formulation). Therefore, the lowest level of meat replacement (5%) already resulted in a significant variation in both L* and b* parameters, resulting in a visibly darker and browner appearance, as shown in Figure 1. Furthermore, the calculated ΔE* values for sausages containing mealworm flour ranged from 7.7 to 9.3. It has been proposed that ΔE* values exceeding three CIELAB units result in color changes distinguishable to the human eye [54].

A similar behavior for the three CIELAB parameters has been reported by Kim et al. [13] in emulsion sausages. A comparable trend was also observed by Choi et al. [39] in frankfurters, although the a* parameter increased with the addition of mealworm flour. A reduction in L* has also been noted in other products, such as biscuits, where wheat was partially replaced with mealworm powder [55]. These color changes are likely due to the presence of inherent melanin, the primary class of pigments in insects, which includes black, brown, and yellow compounds. Additionally, insects can contain carotenoids and flavonoids derived from their diet or other pigments specific to the species [43]. The color of *T. molitor* early larvae is white, progressively turning yellowish-brown in late larve [56], and it is also affected by the different processing techniques applied for flour preparation [57].

### 3.7. Texture Profile

The texture parameters of the different sausages are shown in Table 4. No significant differences were found in any of the evaluated parameters between the control and the reformulated sausages. These results are consistent with findings by Rocchetti et al. [40], who reported no significant changes in texture parameters (hardness, firmness, springiness, cohesiveness, gumminess, and chewiness) in beef burgers fortified with 5% mealworm powder compared to the control. On the other hand, the effect of mealworm flour incorporation on the texture profile has been previously assessed in emulsified sausages, yielding contradictory results. For example, Kim et al. [13] reported an increase in hardness, springiness, and cohesiveness when insect flour was added to the formulation. In contrast, Choi et al. [39] observed a reduction in hardness and chewiness with the addition of insect flour, although these parameters increased when the substitution level exceeded 25%. No differences in cohesiveness or springiness between the different samples was observed, except when 30% insect flour was added [39].

### 3.8. Sensory Analysis

**Figure 2:**
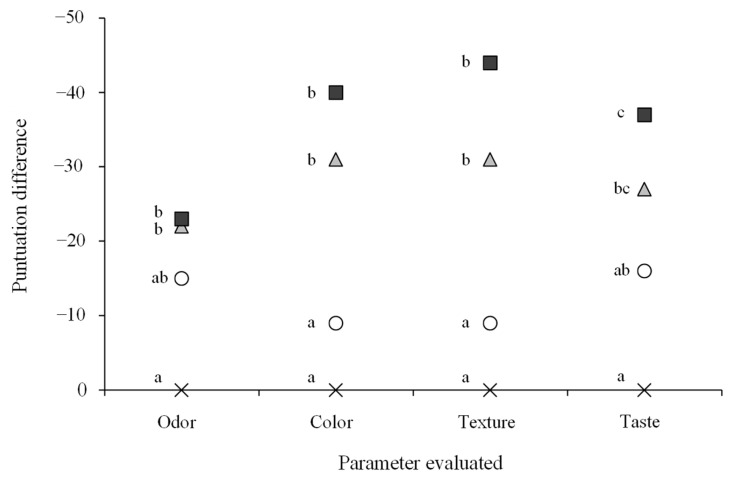
Difference in the sum of ranks of the sensory evaluation between dry fermented sausages manufactured with (○ 5%; ▲ 10%; ■ 15%) and without (×) mealworm flour. a, b, c: different letters in the same parameter indicate significant differences (*p* < 0.05).

The sensory results of dry fermented sausages incorporating mealworm flour compared with the control sample (0% meat replacement) are presented in Figure 2. Replacing 10% or more pork meat with insect flour resulted in significant differences across all evaluated parameters (odor, color, texture, and taste) compared to the control sausages. These findings are consistent with the results of the instrumental color analysis previously described.

Similar outcomes have been reported previously in emulsified sausages, with significant differences observed in the evaluated parameters. Choi et al. [39] found that while concentrations above 5% of mealworm flour caused noticeable changes in color, other sensory attributes (flavor, off-flavor, tenderness, juiciness, and overall acceptability) did not show significant differences until the replacement level exceeded 10%. Similarly, Zhang et al. [15] reported that a low replacement ratio (5%) produced a favorable substitution effect, though this was influenced by the pre-drying treatment applied to the insect flour. In their study, the drying method affected the sensory evaluation, with sausages made using microwave-dried mealworm flour showing better results in terms of inner color, uniformity, and juiciness compared to those made with freeze-dried flour.

Despite the results obtained in our study, which suggest limiting the replacement level to 5%, the findings remain promising. It is important to consider that there are numerous types of fermented sausages, each with distinct characteristics. For instance, some products are made with game meat, such as wild boar, which have a darker color than standard sausages. Others, like the “salchichón de Málaga”, are very soft due to their short maturation time, resulting in lower hardness levels. Additionally, the use of spices such as paprika, oregano, or garlic is common in some products and could help mask any flavors derived from the insect flour. All these factors should be considered when designing future formulations and sensory analyses.

## 4. Conclusions

This study highlights the potential of mealworm flour as a sustainable and nutritionally valuable ingredient in dry fermented sausages, offering a viable alternative to traditional pork-based formulations. The inclusion of mealworm flour enhanced the nutritional profile of the sausages, particularly through increased protein and dietary fiber content and a higher proportion of PUFAs. Remarkably, despite the more prominent unsaturated fatty acid profile, lipid oxidation levels were reduced, probably due to the antioxidant properties of compounds naturally present in the mealworm flour. The antioxidant properties associated with mealworm flour are particularly promising for product reformulation. They could serve as a natural alternative to reduce or replace additives commonly used in meat products to prevent lipid oxidation. Such reformulations could address growing consumer demand for cleaner-label products while maintaining product stability and quality of the final product.

From a sensory perspective, while the incorporation of mealworm flour did not significantly alter the texture, it introduced changes in color, particularly at higher replacement levels (10% and 15%). These changes included a noticeable darkening of the sausages, which, while significant, may be acceptable within the context of the wide diversity of traditional dry fermented meat products. Such products often exhibit considerable variation in color, texture, and flavor due to regional preferences, processing techniques, and ingredient choices. At a lower replacement level (5%), mealworm flour produced sausages with sensory attributes close to the control formulation, making it a promising option for industrial applications that balance nutritional and environmental benefits with consumer acceptability.

Overall, these findings underscore the potential of edible insects as a sustainable protein source capable of reducing the environmental footprint of meat production while enhancing product quality and nutritional value. Future research should focus on exploring the use of mealworm flour in conjunction with spices or other natural additives to improve sensory acceptance and expand its application to food products. Additionally, studies on consumer acceptance and a deeper evaluation of the antioxidant capacity of mealworm flour will be essential to facilitate its broader adoption in the food industry.

## Figures and Tables

**Table 1:** Values of the typical microbiota, pH, a_w_, proximate composition, and lipid oxidation of dry-fermented sausages manufactured with different concentrations of mealworm flour.

	Mealworm Flour (%)				
Parameter	0	5	10	15	*p*-Value
**Typical microbiota (log cfu/g)**					
LAB	8.61 ± 0.39	9.00 ± 0.13	9.06 ± 0.11	9.00 ± 0.14	0.1376
GCC+	7.06 ± 0.41 ^b^	7.40 ± 0.13 ^b^	7.92 ± 0.27 ^a^	8.04 ± 0.36 ^a^	0.0072
*Enterobacteriaceae*	<1.5	<1.5	<1.5	<1.5	-
**pH**	5.26 ± 0.01	5.27 ± 0.09	5.28 ± 0.02	5.37 ± 0.08	0.3905
**a_w_**	0.838 ± 0.016	0.844 ± 0.010	0.854 ± 0.008	0.861 ± 0.014	0.1283
**Composition (g/100 g)**					
Moisture	24.26 ± 0.55 ^c^	25.65 ± 0.22 ^bc^	27.19 ± 0.65 ^ab^	27.96 ± 0.64 ^a^	0.0085
Protein (dry matter)	32.54 ± 0.40 ^b^	33.36 ± 0.35 ^ab^	34.00 ± 0.25 ^ab^	34.62 ± 0.34 ^a^	0.0160
Fat (dry matter)	52.93 ± 0.57 ^a^	51.95 ± 0.28 ^ab^	51.08 ± 0.24 ^b^	50.41 ± 0.35 ^b^	0.0155
Ash (dry matter)	5.51 ± 0.01	5.42 ± 0.08	5.32 ± 0.17	5.25 ± 0.14	0.3289
Total fiber (dry matter)	0.14 ± 0.01 ^d^	0.49 ± 0.03 ^c^	0.78 ± 0.02 ^b^	1.04 ± 0.01 ^a^	0.0000
Carbohydrates (dry matter)	8.87	8.82	8.87	8.86	-
**Lipid oxidation**					
Hydroperoxides (mmol/kg)	51.23 ± 19.23	42.56 ± 17.01	49.75 ± 6.66	38.87 ± 6.62	0.6300
TBARs (mg MDA/kg)	3.91 ± 0.34	3.12 ± 0.91	3.47 ± 0.59	3.32 ± 0.43	0.2106

LAB: lactic acid bacteria; GCC+: Gram-positive catalase-positive cocci. TBARs: thiobarbituric acid-reactive substances. Results are expressed as mean ± standard deviation. ^a,b,c,d^: different letters in the same row indicate significant differences (*p* < 0.05).

**Table 2:** Fatty acid profile (g/100 g FAMES) of the sausages with different concentrations of mealworm flour.

	Mealworm Flour (%)				
FAME	0	5	10	15	*p*-Value
Capric (C10:0)	0.07 ± 0.00	0.07 ± 0.01	0.07 ± 0.00	0.07 ± 0.00	0.5835
Lauric (C12:0)	0.09 ± 0.00	0.10 ± 0.01	0.09 ± 0.01	0.10 ± 0.01	0.8567
Myristoleic (C14:1n-5)	0.02 ± 0.00	0.02 ± 0.00	0.02 ± 0.00	0.02 ± 0.00	0.2422
Myristic (C14:0)	1.15 ± 0.02 ^c^	1.25 ± 0.03 ^b^	1.28 ± 0.00 ^b^	1.38 ± 0.01 ^a^	0.0002
Pentadecanoic (C15:0)	0.07 ± 0.00	0.07 ± 0.00	0.07 ± 0.00	0.07 ± 0.00	0.2036
Palmitoleic (C16:1n-7)	2.20 ± 0.22	2.02 ± 0.03	2.05 ± 0.05	1.94 ± 0.04	0.3302
Palmitic (C16:0)	22.69 ± 0.14	22.54 ± 0.01	22.49 ± 0.06	22.48 ± 0.09	0.1861
Heptadecenoic (C17:1n-7)	0.36 ± 0.00 ^a^	0.35 ± 0.02 ^ab^	0.34 ± 0.01 ^ab^	0.31 ± 0.02 ^b^	0.0331
Heptadecanoic (C17:0)	0.38 ± 0.00 ^a^	0.38 ± 0.02 ^a^	0.37 ± 0.01 ^ab^	0.34 ± 0.00 ^b^	0.0305
Linoleic (C18:2n-6c)	12.25 ± 0.08 ^b^	12.90 ± 0.28 ^a^	13.04 ± 0.13 ^a^	12.99 ± 0.01 ^a^	0.0051
Oleic (C18:1n-9c)	45.69 ± 0.35	45.35 ± 0.51	45.71 ± 0.27	45.64 ± 0.28	0.7355
Stearic (C18:0)	12.35 ± 0.14	12.31 ± 0.23	11.94 ± 0.15	12.13 ± 0.20	0.1588
Nonadecenoic (C19:1n-9)	0.12 ± 0.00 ^a^	0.11 ± 0.00 ^ab^	0.11 ± 0.00 ^ab^	0.10 ± 0.00 ^b^	0.0155
Nonadecanoic (C19:0)	0.05 ± 0.01	0.05 ± 0.00	0.04 ± 0.00	0.05 ± 0.00	0.6618
Arachidonic (C20:4n-6)	0.27 ± 0.01 ^ab^	0.28 ± 0.01 ^a^	0.24 ± 0.00 ^b^	0.24 ± 0.01 ^b^	0.0191
Eicosatrienoic (C20:3n-6)	0.11 ± 0.00	0.11 ± 0.00	0.11 ± 0.00	0.10 ± 0.00	0.4022
Eicosadienoic (C20:2n-6)	0.63 ± 0.02 ^a^	0.60 ± 0.01 ^ab^	0.57 ± 0.00 ^b^	0.56 ± 0.01 ^b^	0.0158
Eicosenoic (C20:1n-9)	1.10 ± 0.02	1.08 ± 0.02	1.06 ± 0.01	1.08 ± 0.03	0.4919
Arachidic (C20:0)	0.23 ± 0.01	0.24 ± 0.01	0.23 ± 0.01	0.25 ± 0.00	0.1253
Docosapentaenoic (C22:5n-3)	0.08 ± 0.01	0.08 ± 0.00	0.07 ± 0.01	0.07 ± 0.00	0.233
Adrenic (C22:4n-6)	0.09 ± 0.00	0.09 ± 0.01	0.08 ± 0.01	0.08 ± 0.00	0.1617
ΣSFA	37.08 ± 0.12	37.00 ± 0.23	36.58 ± 0.21	36.86 ± 0.29	0.1619
ΣUFA	62.92 ± 0.12	63.00 ± 0.23	63.42 ± 0.21	63.14 ± 0.29	0.1449
ΣMUFA	49.48 ± 0.16	48.94 ± 0.51	49.29 ± 0.32	49.10 ± 0.26	0.3366
ΣPUFA	13.44 ± 0.05 ^b^	14.06 ± 0.28 ^a^	14.13 ± 0.11 ^a^	14.05 ± 0.03 ^a^	0.0068
ΣPUFA/ΣSFA	0.36 ± 0.00 ^c^	0.38 ± 0.01 ^b^	0.39 ± 0.00 ^a^	0.38 ± 0.00 ^b^	0.0000
h/H	2.47 ± 0.03	2.49 ± 0.01	2.51 ± 0.01	2.49 ± 0.02	0.3321

FAME: fatty acid methyl ester. SFA: saturated fatty acid. UFA: unsaturated fatty acid. MUFA: monounsaturated fatty acid. PUFA: polyunsaturated fatty acid. Results are expressed as mean ± standard deviation. ^a,b,c^: different letters in the same row indicate significant differences (*p* < 0.05). h/H (hypocholesterolemic/hypercholesterolemic) = (C18:1n-9c + ΣPUFA)/(C12:0 + C14:0 + C16:0).

**Table 3:** Volatile compounds (area units ×10^−5^) identified in the headspace of dry fermented sausages manufactured with different concentrations of mealworm flour.

		Mealworm Flour (%)				
LRI	Compound	0	5	10	15	*p*-Value
	**Amino acid degradation**	1922 ± 95 ^a^	1431 ± 86 ^b^	1274 ± 64 ^b^	1016 ± 73	0.0040
536	Carbon disulfide	142 ± 27	566 ± 40 ^a^	646 ± 62 ^a^	508 ± 70 ^a^	0.0090
654	3-Methylbutanal	666 ± 75 ^a^	183 ± 37 ^b^	98 ± 11 ^b^	72 ± 8 ^b^	0.0004
662	2-Methylbutanal	153 ± 43 ^a^	180 ± 38 ^a^	58 ± 1 ^b^	36 ± 1 ^b^	0.0186
731	3-Methyl-1-butanol	80 ± 9	133 ± 40	144 ± 3	9 ± 5	0.0932
735	2-Methyl-1-butanol	21 ± 0 ^a^	28 ± 0 ^a^	6 ± 2 ^b^	5 ± 0 ^b^	0.0213
739	2-Methyl-2-butenal	143 ± 2	121 ± 37	179 ± 4	111 ± 9	0.0769
842	3-Methylbutanoic acid	53 ± 1 ^c^	173 ± 0 ^a^	121 ± 9 ^b^	171 ± 17 ^a^	0.0008
982	Benzaldehyde	537 ± 25 ^a^	39 ± 3 ^b^	17 ± 2 ^b^	11 ± 2 ^b^	0.0000
1100	Benzeneacetaldehyde	127 ± 0 ^a^	8 ± 4 ^b^	5 ± 1 ^b^	7 ± 1 ^b^	0.0000
	**Carbohydrate fermentation**	10,020 ± 483 ^d^	21,298 ± 1000 ^c^	33,912 ± 852 ^b^	37,950 ± 517 ^a^	0.0000
503	Ethanol	542 ± 64 ^c^	1221 ± 62 ^ab^	1608 ± 132 ^a^	1059 ± 128 ^b^	0.0022
503	2-Propanone (acetone)	141 ± 16 ^b^	186 ± 26 ^b^	341 ± 13 ^a^	148 ± 0 ^a^	0.0008
583	2,3-Butanedione	186 ± 26 ^c^	406 ± 24 ^c^	1617 ± 35 ^b^	2213 ± 115 ^a^	0.0000
604	2-Butanone	369 ± 61 ^c^	744 ± 27 ^b^	1096 ± 96 ^a^	709 ± 28 ^b^	0.0013
649	Acetic acid	6762 ± 467 ^c^	14,588 ± 995 ^b^	20,244 ± 800 ^a^	20,500 ± 437 ^a^	0.0001
711	3-Hydroxy-2-butanone (acetoin)	967 ± 75 ^d^	1977 ± 46 ^c^	7379 ± 235 ^b^	11,370 ± 154 ^a^	0.0000
808	2,3-Butanediol	1053 ± 23 ^c^	2176 ± 46 ^a^	1627 ± 58 ^b^	1951 ± 148 ^a^	0.0007
	**Lipid oxidation**	12,447 ± 169 ^a^	1827 ± 111 ^b^	1630 ± 52 ^b^	1528 ± 86 ^b^	0.0000
	*Aldehydes*	8587 ± 109 ^a^	763 ± 33 ^b^	85 ± 9 ^c^	44 ± 9 ^c^	0.0000
696	Pentanal	1240 ± 66 ^a^	37 ± 11 ^b^	nd	nd	0.0017
825	Hexanal	6124 ± 51 ^a^	627 ± 18 ^b^	67 ± 8 ^c^	32 ± 9 ^c^	0.0000
927	Heptanal	686 ± 63 ^a^	58 ± 21 ^b^	nd	nd	0.0066
989	2-Heptenal	228 ± 10	nd	nd	nd	
1029	Octanal	99 ± 9 ^a^	19 ± 5 ^b^	6 ± 0 ^bc^	4 ± 1 ^c^	0.0002
1091	2-Octenal	58 ± 4	nd	nd	nd	
1131	Nonanal	152 ± 27 ^a^	22 ± 13 ^b^	12 ± 3 ^b^	8 ± 1 ^b^	0.0016
	*Hydrocarbons*	2020 ± 111 ^a^	688 ± 96 ^c^	1151 ± 49 ^b^	1083 ± 58 ^bc^	0.0010
500	Pentane	490 ± 97 ^a^	58 ± 15 ^b^	28 ± 3 ^b^	19 ± 4 ^b^	0.0016
700	Heptane	748 ± 39 ^a^	150 ± 37 ^b^	115 ± 7 ^b^	39 ± 33 ^b^	0.0001
792	1-Octene	64 ± 9 ^a^	35 ± 7 ^b^	47 ± 1 ^ab^	56 ± 3 ^ab^	0.0327
800	Octane	718 ± 37 ^a^	240 ± 54 ^b^	399 ± 44 ^b^	396 ± 5 ^b^	0.0012
992	2,2,4,6,6-Pentamethylheptane	nd	205 ± 68 ^b^	562 ± 20 ^a^	573 ± 47 ^a^	0.0079
	*Alcohols*	1415 ± 62 ^a^	195 ± 12 ^b^	143 ± 13 ^b^	95 ± 6 ^b^	0.0000
512	2-Propanol	45 ± 4 ^b^	52 ± 5 ^ab^	77 ± 12 ^a^	53 ± 6 ^ab^	0.0475
689	1-Penten-3-ol	201 ± 30 ^a^	17 ± 9 ^b^	4 ± 1 ^b^	nd	0.0232
765	1-Pentanol	488 ± 37 ^a^	55 ± 3 ^b^	21 ± 1 ^b^	nd	0.0004
885	1-Hexanol	259 ± 31 ^a^	35 ± 3 ^b^	22 ± 2 ^b^	17 ± 2 ^b^	0.0003
987	1-Heptanol	56 ± 4	nd	nd	nd	-
997	1-Octen-3-ol	324 ± 23 ^a^	25 ± 3 ^b^	13 ± 3 ^b^	14 ± 0 ^b^	0.0000
1089	1-Octanol	42 ± 8	11 ± 1	6 ± 1	11 ± 1	0.2766
	*Ketones*	98 ± 8 ^b^	54 ± 16 ^b^	100 ± 5 ^b^	203 ± 3 ^a^	0.0013
716	2,3-Pentanedione	29 ± 5	28 ± 15	13 ± 2	23 ± 1	0.0328
838	2-Hydroxy-3-pentanone	nd	nd	77 ± 5 ^b^	163 ± 3 ^a^	0.0021
911	2-Heptanone	69 ± 6 ^a^	26 ± 6 ^b^	10 ± 1 ^b^	17 ± 0 ^b^	0.0007
	*Furans*	327 ± 24 ^a^	127 ± 42 ^b^	151 ± 6 ^b^	103 ± 62 ^b^	0.0156
600	2-Methylfuran	57 ± 7	108 ± 42	150 ± 6	102 ± 62	0.2545
701	2-Ethylfuran	147 ± 22 ^a^	17 ± 3 ^b^	nd	nd	0.0140
909	2-n-Butylfuran	8 ± 3 ^a^	2 ± 0 ^b^	1 ± 0 ^b^	1 ± 0 ^b^	0.0133
1009	2-Pentylfuran	115 ± 4	nd	nd	nd	-
	**Microbial esterification**	3729 ± 214	4830 ± 382	5764 ± 629	3834 ± 104	0.1208
615	Ethylacetate	2820 ± 214 ^b^	3799 ± 375 ^ab^	4668 ± 628 ^a^	3044 ± 104 ^b^	0.0274
762	Ethyl 2-methylpropanoate	30 ± 4	68 ± 2	77 ± 29	46 ± 5	0.1459
815	Ethyl butanoate	162 ± 1	126 ± 35	164 ± 4	99 ± 7	0.0592
833	Ethyl 2-hydroxypropionate	35 ± 5	48 ± 22	65 ± 1	26 ± 3	0.0865
862	Ethyl 2-methylbutanoate	122 ± 9	191 ± 55	192 ± 6	150 ± 12	0.1697
867	Ethyl 3-methylbutanoate	342 ± 5 ^c^	525 ± 11 ^a^	530 ± 20 ^a^	410 ± 32 ^b^	0.0016
1012	Ethyl hexanoate	191 ± 4 ^a^	60 ± 13 ^b^	53 ± 1 ^b^	46 ± 2 ^b^	0.0001
1209	Ethyl octanoate	27 ± 1 ^a^	13 ± 0 ^b^	15 ± 2 ^b^	13 ± 1 ^b^	0.0011
	**Spices**	10,549 ± 200	9660 ± 611	10,318 ± 258	9028 ± 334	0.5444
934	α-Pinene	288 ± 60 ^b^	505 ± 70 ^a^	607 ± 2 ^a^	460 ± 13 ^ab^	0.0102
1000	β-Myrcene	234 ± 15 ^a^	168 ± 24 ^ab^	180 ± 21 ^ab^	156 ± 4 ^b^	0.0395
1010	β-Pinene	475 ± 98 ^c^	764 ± 11 ^ab^	892 ± 9 ^a^	651 ± 6 ^bc^	0.0046
1034	3-Carene	4455 ± 43	4255 ± 452	4676 ± 165	4199 ± 19	0.3208
1048	o-Cymene	153 ± 6 ^a^	134 ± 23 ^ab^	127 ± 0 ^ab^	88 ± 6 ^b^	0.0249
1057	Limonene	3794 ± 151 ^a^	2647 ± 366 ^b^	2586 ± 166 ^b^	2388 ± 21 ^b^	0.0091
1065	β-Phellandrene	104 ± 10 ^a^	84 ± 1 ^ab^	72 ± 2 ^b^	67 ± 3 ^b^	0.0068
1084	β-Terpinen	30 ± 8	25 ± 1	24 ± 2	23 ± 0	0.4669
1478	Caryophyllene	1016 ± 43	1075 ± 170	1154 ± 106	996 ± 1	0.4903
	**Total**	38,667 ± 597 ^b^	39,046 ± 1241 ^b^	52,898 ± 1093 ^a^	53,356 ± 541 ^a^	0.0092

nd: not detected. ^a,b,c,d^: values in the same row with different letters are significantly different (*p* < 0.05). LRI: linear retention index on a 5MS/silphenylene polysiloxane column.

**Table 4:** Color (L*, a*, b*, and ΔE*) and texture profile analysis (TPA) parameters of dry fermented sausages manufactured with different concentrations of mealworm flour.

	Mealworm Flour (%)				
Parameter	0	5	10	15	*p*-Value
**Color**					
L*	42.24 ± 5.13 ^a^	36.49 ± 6.82 ^b^	36.12 ± 3.07 ^b^	34.48 ± 2.66 ^b^	0.0013
a*	8.84 ± 2.88	8.99 ± 1.46	7.57 ± 1.48	6.90 ± 1.25	0.0581
b*	5.48 ± 1.19 ^c^	7.52 ± 1.58 ^b^	9.28 ± 1.43 ^a^	9.56 ± 1.58 ^a^	0.0000
ΔE*	-	8.82 ± 2.45	7.74 ± 2.56	9.26 ± 2.32	0.3737
**TPA**					
Hardness (N)	111.47 ± 2.15	95.88 ± 3.89	100.06 ± 11.62	99.04 ± 9.59	0.3422
Adhesiveness (N*s)	−1.62 ± 0.57	−0.99 ± 0.06	−1.91 ± 1.64	−0.78 ± 0.31	0.5921
Springiness	0.71 ± 0.00	0.72 ± 0.00	0.78 ± 0.12	0.74 ± 0.06	0.6989
Cohesiveness	0.45 ± 0.02	0.49 ± 0.00	0.50 ± 0.03	0.50 ± 0.02	0.2064
Chewiness (N)	36.09 ± 1.91	33.52 ± 1.67	37.66 ± 5.75	36.78 ± 4.94	0.8487

Results are expressed as mean ± standard deviation. ^a,b,c^: different letters in the same row indicate significant differences (*p* < 0.05).

## Data Availability

The original contributions presented in this study are included in the article/Supplementary Material. Further inquiries can be directed to the corresponding authors.

## Supplementary Materials

The following supporting information can be downloaded at: https://www.mdpi.com/article/10.3390/foods14061019/s1, Table S1: Formulation of dry fermented sausages manufactured with 0, 5, 10, and 15% pork leg meat replacement by mealworm (*T. molitor*) flour.
