# Supplementary material for: Use of Mealworm (Tenebrio molitor) Flour as Meat Replacer in Dry Fermented Sausages
Source: Foods. 2025 Mar 17;14(6):1019. doi: 10.3390/foods14061019 (PMC11941666; doi:10.3390/foods14061019)
Supplement: Supplementary file 1 [file foods-14-01019-s001.zip › foods-3465274-supplementary.pdf]

Table S1.

Formulation of dry fermented sausages manufactured with 0, 5, 10, and 15% pork leg meat replacement by mealworm (*T. molitor*) flour.

	Mealworm flour (%)			
Ingredients (%)	0	5	10	15
Lean pork	69.944	65.281	60.617	55.954
Pork belly	23.315	23.315	23.314	23.314
Mealworm flour	-	4.663	9.326	13.989
Lactose	2.798	2.798	2.798	2.798
Salt (NaCl)	2.238	2.238	2.238	2.238
Water (ice)	0.933	0.933	0.933	0.933
Glucose	0.466	0.466	0.466	0.466
Black pepper	0.233	0.233	0.233	0.233
Sodium ascorbate	0.050	0.050	0.050	0.050
Sodium nitrite	0.012	0.012	0.012	0.012
Sodium nitrate	0.012	0.012	0.012	0.012
**Total**	100	100	100	100
Additional water[1]	-	3.544	7.088	10.631

[1]Expressed as a percentage over the total 100% formulation. This water is added to compensate for the water removed when reducing the pork meat content. For example, if preparing 1000 grams of total mass and 3.5% additional water is required, 35 grams of water should be added.
